# Omics Analyses of Stromal Cells from ACM Patients Reveal Alterations in Chromatin Organization and Mitochondrial Homeostasis

**DOI:** 10.3390/ijms241210017

**Published:** 2023-06-12

**Authors:** Melania Lippi, Angela Serena Maione, Mattia Chiesa, Gianluca Lorenzo Perrucci, Lara Iengo, Tommaso Sattin, Chiara Cencioni, Matteo Savoia, Andreas M. Zeiher, Fabrizio Tundo, Claudio Tondo, Giulio Pompilio, Elena Sommariva

**Affiliations:** 1 Unit of Vascular Biology and Regenerative Medicine, Centro Cardiologico Monzino IRCCS, 20138 Milan, Italy; mlippi@ccfm.it (M.L.); amaione@ccfm.it (A.S.M.); gperrucci@ccfm.it (G.L.P.); liengo@ccfm.it (L.I.); gpompilio@ccfm.it (G.P.); 2 Department of Medicine and Surgery, Università Degli Studi di Milano Bicocca, 20126 Milan, Italy; 3 Bioinformatics and Artificial Intelligence Facility, Centro Cardiologico Monzino IRCCS, 20138 Milan, Italy; mchiesa@ccfm.it; 4 Department of Electronics, Information and Biomedical Engineering, Politecnico di Milano, 20133 Milan, Italy; 5 Department of Arrhythmology and Electrophysiology, Centro Cardiologico Monzino IRCCS, 20138 Milan, Italy; tomsat90@gmail.com; 6 Istituto di Analisi dei Sistemi ed Informatica “A. Ruberti”, Consiglio Nazionale delle Ricerche (IASI-CNR), 00185 Rome, Italy; chcencioni@gmail.com; 7 Department of Medicine III, Goethe University, Theodor-Stern-Kai 7, 60590 Frankfurt am Main, Germany; savomat88@gmail.com (M.S.); zeiher@em.uni-frankfurt.de (A.M.Z.); 8 Heart Rhythm Center, Centro Cardiologico Monzino IRCCS, 20138 Milan, Italy; fabrizio.tundo@ccfm.it (F.T.); ctondo@ccfm.it (C.T.); 9 Department of Biomedical, Surgical and Dental Sciences, Università degli Studi di Milano, 20122 Milan, Italy

**Keywords:** arrhythmogenic cardiomyopathy, cardiac mesenchymal stromal cells, omics, methylome, transcriptome, mitochondria, chromatin, epithelial-to-mesenchymal transition, proliferation

## Abstract

Arrhythmogenic cardiomyopathy (ACM) is a genetic disorder characterized by ventricular arrhythmias, contractile dysfunctions and fibro-adipose replacement of myocardium. Cardiac mesenchymal stromal cells (CMSCs) participate in disease pathogenesis by differentiating towards adipocytes and myofibroblasts. Some altered pathways in ACM are known, but many are yet to be discovered. We aimed to enrich the understanding of ACM pathogenesis by comparing epigenetic and gene expression profiles of ACM-CMSCs with healthy control (HC)-CMSCs. Methylome analysis identified 74 differentially methylated nucleotides, most of them located on the mitochondrial genome. Transcriptome analysis revealed 327 genes that were more expressed and 202 genes that were less expressed in ACM- vs. HC-CMSCs. Among these, genes implicated in mitochondrial respiration and in epithelial-to-mesenchymal transition were more expressed, and cell cycle genes were less expressed in ACM- vs. HC-CMSCs. Through enrichment and gene network analyses, we identified differentially regulated pathways, some of which never associated with ACM, including mitochondrial functioning and chromatin organization, both in line with methylome results. Functional validations confirmed that ACM-CMSCs exhibited higher amounts of active mitochondria and ROS production, a lower proliferation rate and a more pronounced epicardial-to-mesenchymal transition compared to the controls. In conclusion, ACM-CMSC-omics revealed some additional altered molecular pathways, relevant in disease pathogenesis, which may constitute novel targets for specific therapies.

## 1. Introduction

Arrhythmogenic cardiomyopathy (ACM) is a rare inherited disorder of the heart, presenting incomplete penetrance and variable expressivity. In many cases, ACM patients display malignant arrhythmias and progressive contractile dysfunctions, which can lead to sudden cardiac death and heart failure, respectively. ACM ventricular myocardium, mainly in the right ventricle, is characterized by wide cardiomyocyte loss, inflammation and fibro-adipose tissue replacement, with consequent electrical instability and mechanical impairment, whose progression follows an epicardium–endocardium gradient [1]. ACM mainly affects young people, especially males, who are more severely and frequently affected than females, despite the autosomal inheritance [2]. About 50% of patients carry a disease-causing mutation, which is found mainly in genes encoding desmosomal proteins, including prevalently plakophilin-2 (*PKP2*), but also plakoglobin (*JUP*), desmoplakin (*DSP*), desmoglein-2 (*DSG2*) and desmocollin-2 (*DSC2*) [3,4]. Dysfunctions in desmosomes alter mechanical stability, electrical coupling between cells and cellular signaling [5,6,7]. Less frequently, variants in nondesmosomal genes are associated with ACM, such as *TMEM43*, *PLN* and *DES* [4,8,9,10,11].

The complex ACM phenotype is the result of the contribution of different cardiac cell types. Cardiomyocytes (CM) are mainly responsible for electrical and contractile impairments, while cardiac mesenchymal stromal cells (CMSCs) are responsible for fibro-adipose substitution, which worsens the dysregulation of functional and electrical characteristics [12,13,14]. CMSCs are an abundant cardiac cell population with an epicardial origin [15], a differentiation potency toward adipocyte and fibroblast fate [16,17], and a crucial role in cardiac homeostasis and remodeling during pathological conditions [18]. In addition, since CMSCs express desmosomal proteins and develop the fibro-fatty phenotype in vitro, these cells are a valid model to study ACM and to perform mechanistic studies [12,13].

Previous basic and translational research focused on the comprehension of molecular mechanisms driving ACM phenotype. Among these, some studies demonstrated the alteration of the Wnt pathway, which normally induces proliferation and cell fate specification thanks to the interaction of β-catenin with specific transcription factors [19]. The abnormal nuclear localization of plakoglobin protein (PG) found in ACM patients provokes the competition between PG and β-catenin, causing a detrimental effect on Wnt signaling and ultimately activating adipogenesis, fibrosis and apoptosis [12,20].

Another dysregulated pathway in ACM is the Hippo pathway, which regulates cell proliferation, apoptosis and cell fate, and responds to mechanical stimuli or cell–cell contacts. The aberrant phosphorylation of Yes-associated protein (YAP), which is the Hippo pathway effector, inhibits YAP canonical transcriptional function, limiting cellular proliferation in ACM-HL-1 myocytes. Moreover, YAP inhibits Wnt signaling through the interaction with β-catenin, which is prevented from the nuclear localization, promoting adipogenesis [7].

A loss of PKP2 alters the macromolecular complex of which the protein is part, along with the gap-junction protein connexin 43 and the voltage-gated sodium channel Nav1.5. This causes a remodeling of the intercalated disc structure and a reduction in sodium current [21,22].

A contribution to ACM pathogenesis is also given by modifications in intracellular calcium homeostasis. ACM-related mutations in *PLN*, encoding the Ca^2+^ pump regulator, exert a deleterious effect on calcium-handling machinery, thus triggering apoptosis, electrical instability and lipid accumulation [8]. Furthermore, it has recently been demonstrated that ACM-CMSCs present an increased frequency of spontaneous Ca^2+^ oscillations, which, activating CaMKII, contribute to the ACM aberrant lipid/fibrotic accumulation [23].

The previously cited researches, including a first attempt of characterization of ACM-CMSCs [24], provided useful clues on the pathogenic mechanism occurring in ACM, even if a full understanding is still missing. In particular, a complete characterization of the molecular pathways underlying stromal cell involvement is needed.

In order to contribute to this scope, we performed and validated analyses of gene expression coupled to an epigenetic profiling of CMSCs obtained from ACM patients and healthy subjects, for the characterization of CMSC-specific ACM pathogenic molecular mechanisms and for the identification of novel targets for therapeutic interventions.

## 2. Results

### 2.1. Methylome Analysis

**Figure 1:**
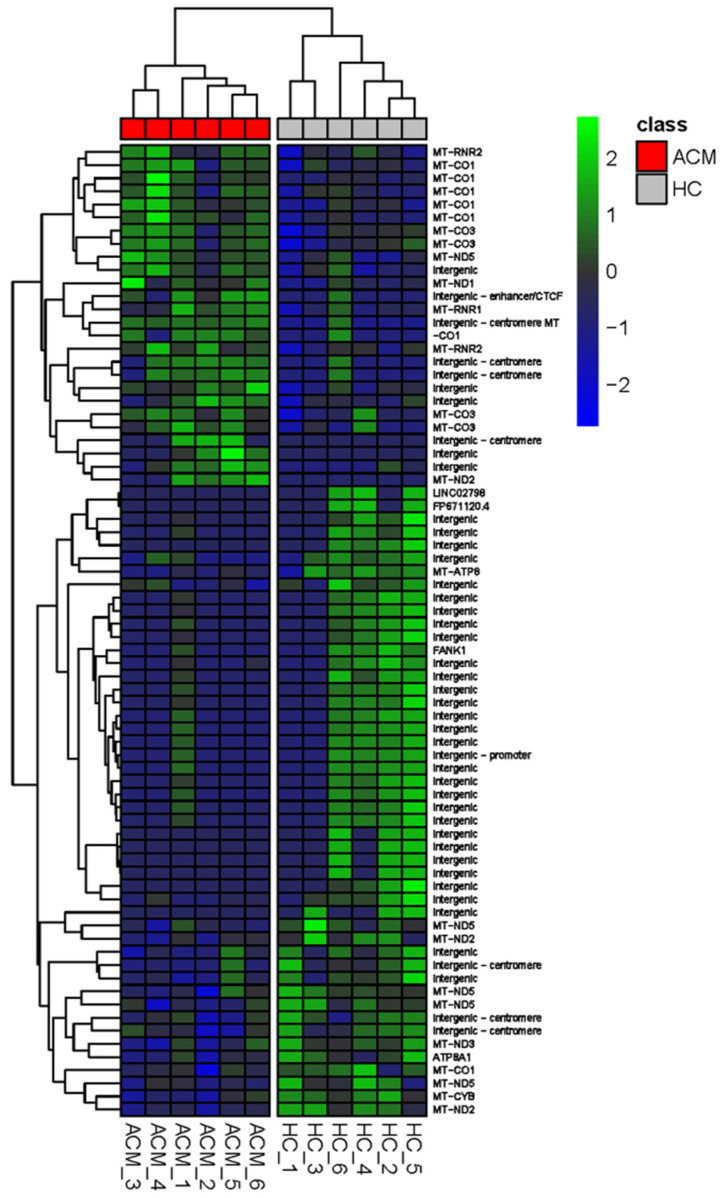
Methylome analysis shows the differential methylation of 74 nucleotides in arrhythmogenic cardiomyopathy (ACM)- vs. healthy control (HC)-cardiac mesenchymal stromal cells (CMSCs). Heatmap showing the significant differentially methylated bases in ACM vs. HC samples, whose log of fold change (logFC) is represented by the color coding shown in the legend (in green, higher DNA methylation levels in ACM-CMSCs vs. HC-CMSCs; in blue, lower DNA methylation levels in ACM-CMSCs vs. HC-CMSCs).

With the aim to compare the DNA methylation profile of CMSCs from ACM and healthy subjects, we carried out a methylome analysis on six ACM and six HC samples. Among the 12,466 methylated bases, we found that the methylation of 74 nucleotides was significantly different in ACM-CMSCs compared to the controls (*p*-value ≤ 0.05) (Figure 1; Table S1). Interestingly, 30 differentially methylated bases were located on the mitochondrial chromosome, 28 of which were in coding genes and 2 in intergenic regions. Most of the other bases of interest were on repeated sequences in intergenic regions of the nuclear chromosomes, including the centromere. In particular, we found a region containing repetitive elements in chromosome 1 and two regions in chromosome 21, where DNA is less methylated in ACM-CMSCs compared to controls (Table S2). The heatmap in Figure 1 illustrates that the significant differences in methylation of the DNA nucleotides are consistent in the distinct ACM vs. HC samples, as they enabled unsupervised clustering of ACM and HC groups.

### 2.2. Transcriptome Analysis

**Figure 2:**
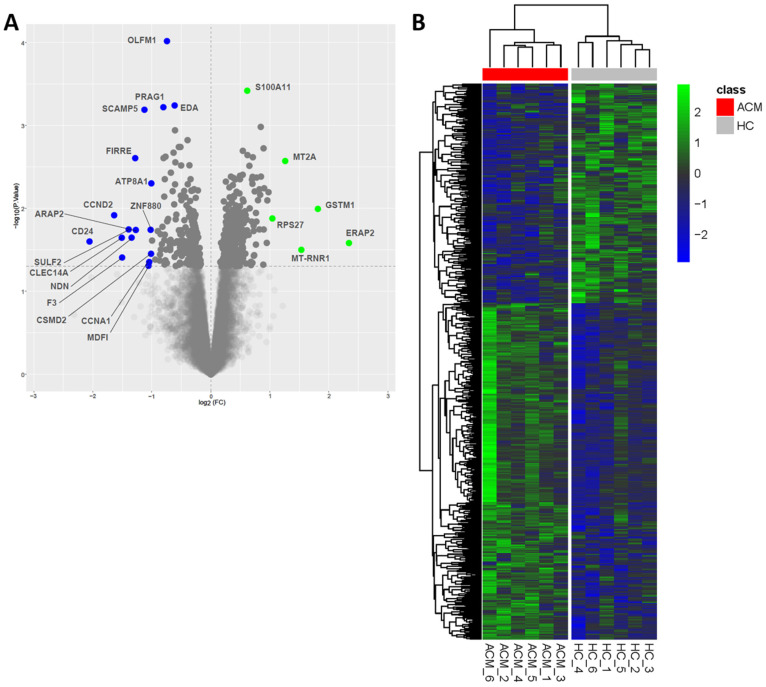
Transcriptome analysis reveals the differential expression of 529 genes in ACM- vs. HC-CMSCs. Volcano plot showing the extent (log2 of Fold Change, *x*-axis) and significance (−log10 of *p*-values, *y*-axis) of differential expression between ACM and HC samples for each gene. The horizontal dashed line represents the cut-off value used to define differentially expressed genes; most differentially expressed genes have been highlighted in blue (*p*-value < 0.01; logFC < −1) and green (*p*-value < 0.01; logFC > 1) (**A**). Heatmap showing the differentially expressed genes, whose logFC values are represented by the color coding shown in the legend (in green, genes more expressed in ACM-CMSCs vs. HC-CMSCs; in blue, genes less expressed in ACM-CMSCs vs. HC-CMSCs) (**B**).

In order to define changes in the gene expression profiles between ACM- and HC-CMSCs, we performed a high-throughput RNA-seq on the same six ACM and six HC samples used for methylome analysis. Out of 15,031 expressed genes, 529 were differentially expressed (*p*-value ≤ 0.05) with a log of fold change (logFC) ranging from −2.06 to 2.34 (Table S2). In particular, 327 were more expressed in ACM vs. the control CMSCs, whereas 202 were less expressed (Figure 2A). The heatmap in Figure 2B shows the differentially expressed genes that allowed the clear clustering of ACM and HC samples.

Among the most differentially expressed genes (*p*-value < 0.01; logFC < −1 or logFC > 1; Figure 2A), we focused on those involved in pathways with potential higher relevance in the pathogenesis of ACM, and never explored in stromal cells in depth.

*CCND2*, coding for cyclin2D and directly involved in the progression of the cell cycle, is less expressed in ACM compared to the controls. Similarly, other genes promoting the cell cycle, such as *CCNA1*, *CCNG2*, *CCNB1IP1*, *CDK5RAP2*, *CDK19*, *CDK11B*, *CDK16*, *CDK6*, *CDK5R*, *CDK4* and *CDK5*, showed the same trend. These findings suggest that ACM-CMSCs might exhibit a lower proliferation rate than HC-CMSCs.

The higher expression in ACM vs. HC cells of the *COX7C* gene, implicated in mitochondrial respiration, is in line with the methylome results. Other genes involved in mitochondria activity are mildly more expressed in ACM than in HC, such as *COX15*, *MT-CO2*, *MT-ND3*, *MT-ND1*, *MT-CYB*, *MT-ATP8*, *MT-ND4L* and *MT-RNR1*. It is therefore likely that the normal mitochondrial functioning in ACM-CMSCs is disturbed.

*S100A11* is involved in Wnt/β-catenin signaling and epicardial-to-mesenchymal transition (EMT). The transcriptomic results revealed that it is highly expressed in ACM-CMSCs. Accordingly, some epicardial markers are less expressed in ACM samples with respect to controls, such as *ALDH1A1*, *TCF21* and *WT1*. This suggests that EMT could be more pronounced in ACM-CMSCs than in the control samples.

### 2.3. Enrichment and Gene Network Analysis

**Figure 3:**
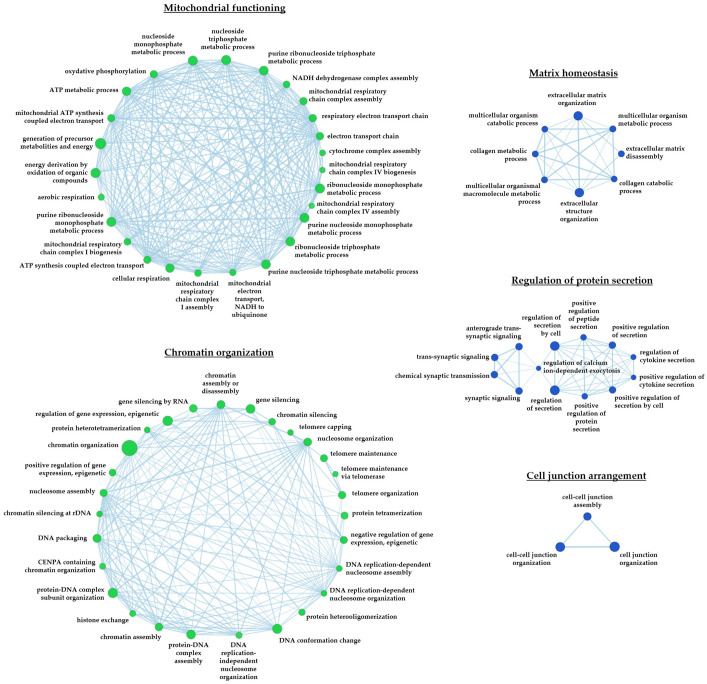
Known and novel altered pathways in ACM-CMSCs. Functional network of differentially expressed genes in CMSCs. Nodes represent gene groups implicated in common biological processes. Green nodes indicate pathways more expressed in ACM than HC; blue less expressed. The larger the nodes, the more genes are regulated in the node.

We performed an enrichment and a gene network analysis, building functional networks, where connected nodes represent gene groups implicated in common biological processes. We found 315 gene groups differentially regulated in ACM- and HC-CMSCs (Table S3). Some of these are implicated in pathways known to be altered in ACM, such as matrix homeostasis (eight gene groups) and cell junction arrangement (three gene groups), while other concerned pathways never associated with ACM to our knowledge. Among these, 26 gene groups were implicated in mitochondria homeostasis and 28 in chromatin organization, and both findings are in line with the methylome results. In addition, protein secretion (13 nodes) was impaired in ACM (Figure 3).

### 2.4. Validation at Protein Level

**Figure 4:**
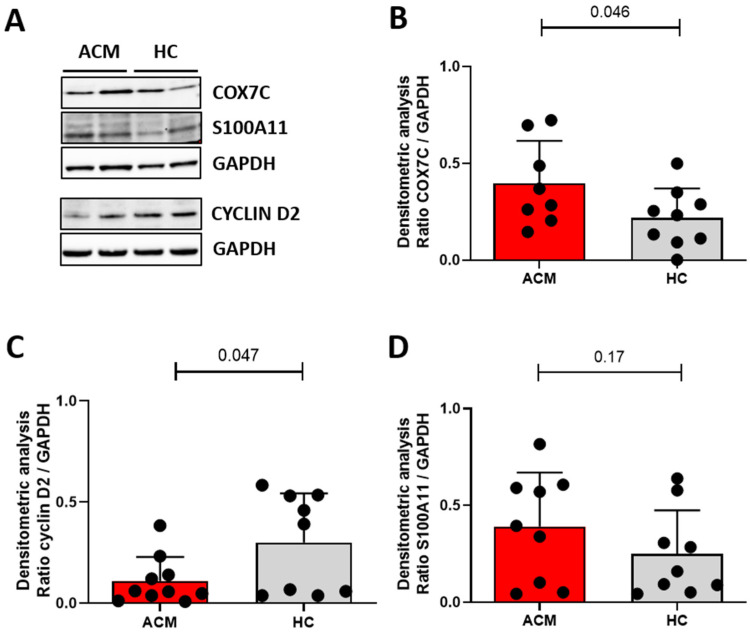
Western blot analysis confirms the differential expression of COX7C, cyclin D2 and S100A11 at protein levels between ACM- and HC-CMSCs. Representative immunoblots (**A**) and relative quantification with graph bars of differential protein levels of COX7C (**B**), cyclin D2 (**C**) and S100A11 (**D**), normalized on GAPDH levels. All data are shown as mean ± standard deviation.

To validate the main transcriptome findings, we quantified the expression of proteins coded by *COX7C* and *S100A11*, which are more expressed in ACM than HC, and *CCND2*, which is less expressed in ACM than HC. The Western blot analysis shown in Figure 4 demonstrated that the protein levels of COX7C and S100A11 are higher in ACM- than in HC-CMSCs (COX7C: 0.397 ± 0.219 in ACM vs. 0.219 ± 0.151 in HC, n = 8 ACM vs. 9 HC, *p*-value = 0.046; S100A11: 0.391 ± 0.279 in ACM vs. 0.250 ± 0.225 in HC, n = 9 each, *p*-value = 0.17). Likewise, cyclin D2 expression was lower in ACM- compared to HC-CMSCs, in according with transcriptome (0.12 ± 0.11 in ACM vs. 0.3 ± 0.243 in HC, n = 10 ACM vs. 9 HC, *p*-value = 0.047).

### 2.5. Quantification of Active Mitochondrial and ROS Production

**Figure 5:**
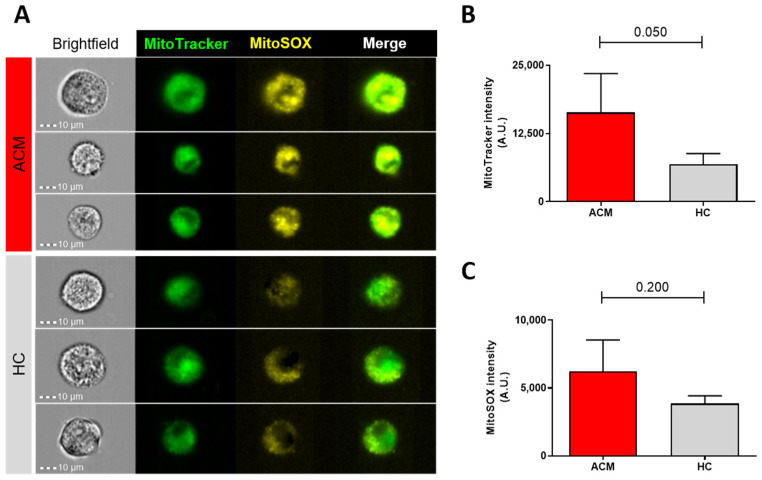
MitoTracker and MitoSOX staining reveals mitochondrial dysfunctions in ACM-CMSCs compared to controls. Representative ImageStreamX images of ACM and HC CMSCs in brightfield (first column), labeled with MitoTracker (second column, in green), with MitoSOX (third column, in yellow) and the merge of the two fluorescent signals (fourth column) (**A**). Graph bars indicating the intensity of fluorescence of MitoTracker- (**B**) and MitoSOX-positive CMSCs (**C**) in ACM (red bars) vs. HC (grey bars). All data are shown as mean ± standard deviation.

The above mentioned results support the presence of mitochondrial dysfunctions in ACM-CMSCs. As a preliminary confirmation, we quantified the active mitochondrial amount and evaluated mitochondrial ROS production in ACM- and HC-CMSCs. The analysis at ImageStreamX shown in Figure 5 revealed that ACM-CMSCs have a higher fluorescence intensity for both MitoTracker (marker of active mitochondria) and MitoSox (marker of mitochondrial ROS) compared to control cells. (MitoTracker: 16,345.8 ± 7152 in ACM vs. 6835.0 ± 1995 in HC, n = 3 each, *p*-value = 0.050; MitoSOX: 6235.5 ± 2312 in ACM vs. 3851.0 ± 579 in HC, n = 3 each, *p*-value = 0.200).

### 2.6. Proliferation Analysis

**Figure 6:**
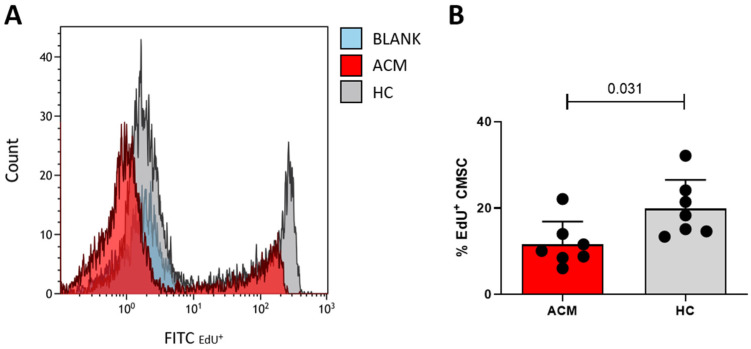
EdU assay shows the lower proliferation rate of ACM- vs. HC-CMSCs. The representative histogram shows the differences in EdU incorporation between ACM- and HC-CMSCs (**A**). Graph bars indicate the percentage of EdU-positive CMSCs in ACM (red bars) vs. HC (grey bars) samples, obtained through FACS analysis (**B**).

Given the low expression in ACM cells (transcriptome data) of the cell cycle regulator *CCND2*, which promotes cell proliferation, we tested if ACM-CMSCs showed a lower proliferation rate than controls by 5-ethynyl-20-deoxyuridine (EdU) assay. As shown in Figure 6, we found that a lower number of ACM-CMSCs incorporate EdU in the DNA compared to HC cells in the same time period (4 h). This indicates that ACM-CMSCs proliferate less (11.60 ± 5.27% in ACM vs. 19.89% ± 6.65 in HC, n = 7 each, *p*-value = 0.031).

### 2.7. Epicardial-to-Mesenchymal Transition

**Figure 7:**
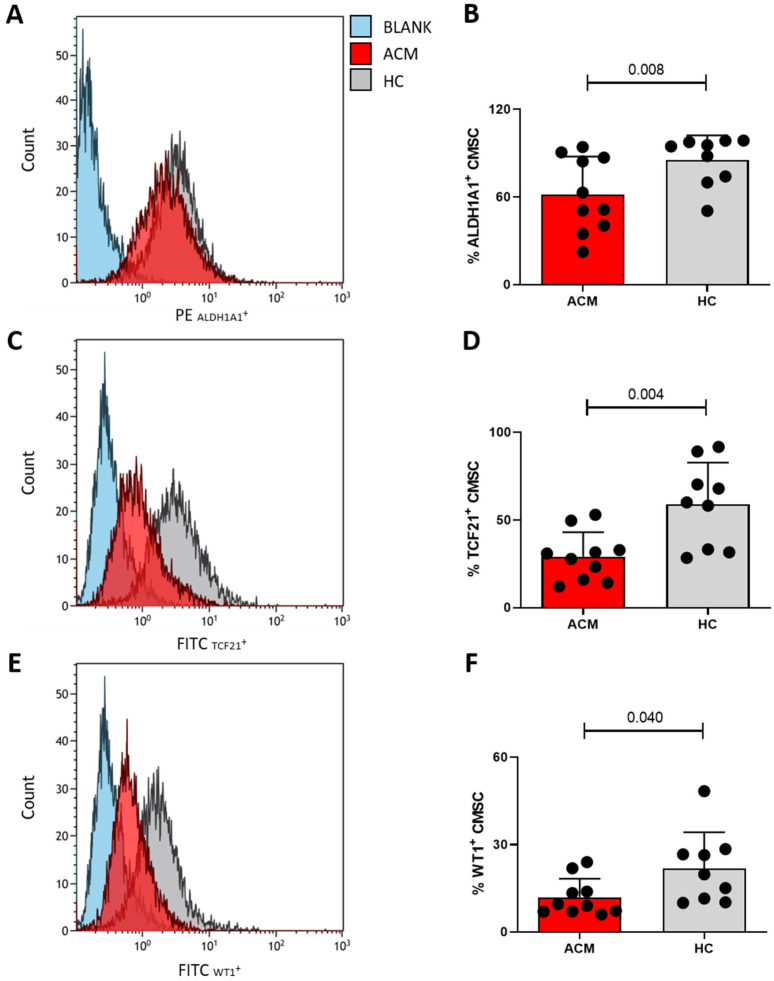
FACS analysis demonstrated a lower expression of epicardial markers at protein levels in ACM-CMSCs compared to controls. The representative histogram and the relative graph bars indicate the percentage of ACM- vs. HC-CMSCs, positive for ALDH1A1 (**A**,**B**), positive for TCF21 (**C**,**D**) and positive for WT1 (**E**,**F**) obtained through FACS analysis. All data are shown as mean ± standard deviation.

The transcriptomic results suggested that EMT in ACM-CMSCs might be more pronounced than in HC-CMSCs. We verified this transcriptomic result by measuring the protein expression of the epicardial markers aldehyde dehydrogenase 1A1 (ALDH1A1), transcription factor 21 (TCF21) and Wilms Tumor Protein (WT1) in CMSCs by FACS analysis. We found that ACM cells exhibited a minor expression of epicardial markers compared to HC (Figure 7). (ALDH1A1: 61.9% ± 25.81 in ACM vs. 85.3% ± 16.89 in HC, n = 10 ACM vs. 9 HC, *p*-value = 0.008; TCF21: 29.2% ± 13.89 in ACM vs. 59.0% ± 23.81 in HC, n = 10 ACM vs. 9 HC, *p*-value = 0.004; WT1: 11.9% ± 6.41 in ACM vs. 21.8% ± 12.38 in HC, n = 10 ACM vs. 9 HC, *p*-value = 0.040).

## 3. Discussion

In the present study, we carried out a hypothesis-free approach based on the DNA methylation and gene expression profile of CMSCs derived from ACM patients and HC, in order to detect novel factors involved in the pathogenesis of the disease. We detected and preliminarily validated some dysregulated pathways never identified in ACM-CMSCs to date.

The variable expressivity of ACM suggests that epigenetic factors could influence the phenotype of the disease. For this reason, we drew a preliminary epigenetic profile of ACM-CMSCs by performing a methylome analysis. Indeed, we noted three clusters of lower methylated basis in ACM- vs. HC-CMSCs in chromosomes 1 and 21. These are regions with repetitive elements, whose correct methylation status is essential to avoid genomic instability, which might potentially represent a source of DNA mutations and damage. Recently, Pérez-Hernández et al. demonstrated that *PKP2*-knockout cardiomyocytes are characterized by chromatin disorganization and DNA damage, started by the nuclear envelope disruption due to PKP2 loss [25]. We speculate that this condition could involve an altered DNA methylation in ACM cells. In addition, the alteration of the chromatin organization, emerged in the enrichment and gene network analyses of the transcriptome, suggests that other epigenetic mechanisms could be involved in ACM pathogenesis. In particular, among the genes involved in chromatin arrangement, the transcriptome analysis highlighted the differential expression of the genes *HIST3H2BB* and *MBD2* (Table S1). *HIST3H2BB* encodes a replication-dependent histone, a member of the histone H2B family, whose regulation could constitute a potential epigenetic therapeutic strategy [26]. *MBD2* encodes methyl-CpG-binding domain protein 2, which mediates the molecular consequences of the DNA methylation [27]. Chemical probes targeting MBD2 have already been reported in the literature, and it is possible that these compounds could become an upcoming treatment for ACM [28].

In our work, multiple results converge to confirm that a mitochondrial dysfunction is present in ACM stromal cells, starting from the significant differences in mitochondrial DNA methylation, to the transcriptomic results, the network analysis and the preliminary functional studies. Previously, other authors hypothesized the role of mitochondria in the electrical instability of ACM cardiomyocytes, postulating that metabolic and mitochondrial impairments could constitute substrates for electrical and structural remodeling in ACM hearts, but the theory has not been tested yet, to our knowledge [29]. By our methylome analysis, we found half of the differentially methylated bases on coding genes located on mitochondria chromosome. Information concerning the link between “mitoepigenetics” and human diseases is only lately emerging, and the biological function of mitochondrial DNA methylation has been debated in recent years [30]. However, evidence of the impact of an altered methylation status on the mitochondrial chromosome has been reported in the context of cancer and neurodegenerative, metabolic and cardiovascular diseases [30,31]. With our data, we can speculate that the different fluorescent intensity of the MitoTracker probe in ACM- and HC-CMSCs could be due to the different metabolic status. It has been reported in the literature that mesenchymal cells change their metabolic activity during the differentiation: the undifferentiated cells depend more on glycolysis than differentiated offspring cells, which instead depend more on oxidative phosphorylation [32,33,34,35]. It is likely that the greater propensity of ACM-CMSCs to differentiate, as previously demonstrated [12,13], is also reflected in a higher state of mitochondrial activation. This high activity could be due either to the increase in mitochondria number, whose self-renewal biogenesis has been observed during adipogenesis in mesenchymal cells [36]; and/or to the increase in the mitochondrial membrane potential, which mirrors the metabolic profile of precommitted mesenchymal cells [37]. Our results are in line with a recent study that detected copious mitochondrial clustering in cardiomyocytes from right ventricular biopsy derived from an ACM patient and observed higher levels of oxidant generation in *PKP2*-knockout cardiomyocytes compared to the controls [25].

We also detected a higher fluorescent intensity of the MitoSOX probe in ACM-CMSCs, which could be merely due to an increased mitochondrial activity or to an impairment in the mitochondrial respiratory chain, which generates and leaks ROS. In our laboratory, we previously demonstrated that the oxidative stress represents a cofactor contributing to the pathogenesis of ACM, correlating with the severity of the symptomatology [38]. It is likely that mitochondrial dysfunction is a contributory cause of oxidative stress in ACM. More in-depth studies are needed to identify the specific mitochondrial processes that are altered, to understand whether these differences are a cause or a consequence of the cardiomyopathy or both, and to understand whether they could constitute a pharmacological target to attenuate or to delay patients’ symptomatology.

Through transcriptome and Western blot analyses, we found in ACM- vs. HC-CMSCs a lower expression of *CCND2* and its coded protein cyclin D2, whose binding with cyclin-dependent kinases 4 and 6 promotes cell proliferation [39]. Our functional study, carried out by examining the incorporation of EdU during DNA synthesis, showed a lower cell division rate in ACM-CMSC, according to the gene and protein expression levels. Since generally cells maintain a balance between self-renewal and differentiation, we hypothesized that a lower proliferation rate may correspond to a higher propensity of ACM-CMSC to differentiate. In addition, different perturbations of the Wnt/β-catenin pathway in ACM are known and can negatively regulate the cell cycle [19]. Furthermore, in the enrichment and gene network analysis, we found that the pathway concerning the regulation of cell aging is more expressed in ACM samples vs. HC, as well as the pathways involved in the telomere capping, maintenance and organization (Table S3). It is possible that cell cycle dynamics changes are linked to premature senescence in ACM-CMSCs.

Another interesting result obtained by our analysis is a marked EMT in ACM-CMSCs, suggested by the higher expression of *S100A11* in the transcriptome, compared to HC. The product of *S100A11* gene is a calcium-binding protein that enhances epithelial-to-mesenchymal transition in cancer [40,41]. Epithelial-to-mesenchymal transition is induced by transforming growth factor β1 (TGF-β1) [42], and this is in line with the previous demonstration of TGF-β1’s high expression in ACM [13]. The lower protein expression of epicardial markers ALDH1A1, TCF21 and WT1 in ACM CMSCs compared to the controls confirmed our hypothesis. Indeed, since the loss of junction proteins is one of the first steps of EMT, ACM cells, generally expressing lower amounts of PKP2 protein, are expected to undergo more EMT [12]. Different authors previously reported that EMT underlies the fibro-adipose phenotype in ACM [43,44]. In particular, Kohela et al. demonstrated that ACM epicardial cells, derived from human-induced pluripotent stem cells, spontaneously differentiate into fibro-fatty cells through an enhanced EMT. Furthermore, they observed the activation of the epicardium in ACM explanted hearts [43]. The response of the epicardium to acute cardiac injury is known to start the EMT to generate subepicardial fibroblasts in a Wnt/β-catenin-dependent manner [45,46]. In ACM, it is likely that cardiomyocyte loss/damage constitutes one of the triggers driving EMT. Considering the known progressive fibro-adipose substitution from the epicardium to the endocardium and the known epicardial origin of CMSC, these results suggest that in the fibro-adipose remodeling, a crucial role is played by EMT, which we established to be more enhanced in ACM-CMSC compared to controls [1,15].

Some of the pathways that emerged from our analyses were in line with the available literature about ACM pathogenesis: (i) the network involving the matrix homeostasis, whose dysfunction is well established in the fibrotic replacement that ACM hearts undergo [13]; (ii) the network involving the cell junction arrangement, on which the Hippo and Wnt/β-catenin pathways are known to depend [47,48].

Similarly to what we described, previous transcriptome analyses on *PKP2*-mutated cardiac samples (both bulk and single-cell sequencing) revealed a significant modulation of the genes involved in mitochondrial membrane and in the extracellular matrix regulation [49,50].

In conclusion, in the present study, we provided some pieces of the complex molecular puzzle that builds the ACM pathogenesis. We focused on the mechanisms underlying CMSC behavior, through omics analyses and functional validations, which revealed the potential role of epigenetic, mitochondrial and proliferation dysfunctions in addition to already known pathogenetic pathways. These molecular pathways may hold the keys to the discovery of new druggable therapeutic targets with the aim to improve the clinical management of ACM, both to alleviate symptomatology and to slow disease progression.

## 4. Materials and Methods

### 4.1. ACM Population

This study complies with the declaration of Helsinki and was approved by the Istituto Europeo di Oncologia and Centro Cardiologico Monzino Ethics Committee (R1020/19-CCM1072; date of approval: 3 July 2019). Different CMSCs were used for the in vitro experiments depending on availability and culture passage number. The studied population included 14 unrelated ACM patients fulfilling the 2010 International Task Force Criteria [51] or ACM diagnostic Padua Criteria [52], recruited at Centro Cardiologico Monzino IRCCS from 2014 to 2020. A concise table summarizing the clinical characterization of each patient is consultable in Supplementary Materials (Table S4).

### 4.2. CMSC Isolation and Culture

Cells were obtained through the digestion of endomyocardial biopsies, and they were characterized as previously described [53]. The culture medium for CMSC maintenance was Iscove’s Modified Dulbecco’s Medium (Thermo Fisher Scientific, Waltham, MA, USA), supplemented with 20% fetal bovine serum (EuroClone, Pero, Italy), 10 ng/mL basic fibroblast growth factor (Peprotech, London, UK), 10,000 U/mL Penicillin (EuroClone), 10,000 μg/mL Streptomycin (EuroClone) and 0.02 M L-Glutamine (EuroClone). All the assays were performed on CMSCs cultured in these conditions.

### 4.3. Methylome Analysis

Patients’ DNA was extracted through QIAamp DNA Kit Mini (QIAGEN, Hilden, Germany) following the recommended instructions. DNA was extracted from 6 ACM and 6 control CMSCs, and methylome analyses were performed by bisulfite conversion of DNA combined with next-generation sequencing (NGS), using the Ovation Ultralow Methyl-Seq DR Multiplex system method (performed by GenomeScan, based on Agilent Technologies, Santa Clara, CA, USA). Briefly, after sodium bisulfite treatment, unmethylated cytosines in single-stranded DNA were deaminated to give uracil while leaving methylated cytosine intact. Next, bisulfite-converted DNA were analyzed by NGS. The depths of necessary sequencing were obtained using the Illumina HiSeq4000 platform (Illumina, San Diego, CA, USA).

The Bismark tool [54] was used to (1) align reads against the GRCh38 Human Genome reference (version 99), (2) identify the methylation sites and (3) assess the methylation level. Then, we generated a consensus list of 12,796 methylations, and performed the differential analysis.

### 4.4. Transcriptome Analysis

For sequencing via Illumina HiSeq (50–100 Million reads/sample), we extracted total RNA of CMSCs from the same 6 ACM and 6 control subjects as for the methylome, using the total RNA purification kit (Norgen Biotek Corp., Thorold, ON, Canada) and following the recommended protocol. High-quality libraries were prepared using the PrepXTM RNA-Seq Library Kit for Illumina platforms. Sequential aligning of raw reads was performed against the GRCh38 Human Genome reference (version 96) with ‘STAR’ [55] and ‘Bowtie 2’ [56] to locally align any reads not mapped by STAR. Gene expression quantification and annotation was computed by “featureCounts” [57]. Then, when fewer than 10 reads aligned in at least 40% of the gene, that gene was deemed as not expressed. The expressed genes were further normalized (variance stabilizing normalization) by the ‘DaMiRseq R package [58]. Finally, the differential analysis (ACM vs. HC) was performed by the ‘limma’ R package [59].

### 4.5. Enrichment and Gene Network Analysis

To infer the biological functions associated with the ACM phenotype, we exploited gene set enrichment analysis (GSEA; v2.2) using the Gene Ontology (GO) Biological Processes (BP) repository as reference of prior biological knowledge [60]. GSEA parameters were not modified, except for the number of permutations (set to 10,000) and the number of evaluated gene sets; specifically, genes sets with a number of associated genes ranging from 15 to 500 were selected. GSEA results were represented as a network by Enrichment Map (v3.3.4) [61], a plug-in of Cytoscape v3.9.1 [62], after selecting only significant pathways (FDR-adjusted *p*-value < 0.05).

### 4.6. Protein Extraction and Western Blot Analysis

We evaluated the levels of specific proteins selected from transcriptomic analysis on ACM and control CMSCs. Cells were lysed in cell lysis buffer (Cell Signaling Technology, Danvers, MA, USA), with the addition of protease and phosphatase inhibitors (Sigma-Aldrich, Saint Louis, MO, USA). After the run into Tris-Glycine or NuPage Bis-Tris gels (Sigma-Aldrich), total protein extracts were transferred onto a nitrocellulose membrane (Bio-Rad, Hercules, CA, USA). The membranes’ blocking occurred in 5% nonfat dry milk in wash buffer (PBS, 0.1% Tween-20) and the incubation with the proper primary antibodies (Table S5) against cyclin D2 (CCND2; Abcam, Cambridge, UK), Cytochrome c Oxidase 7C (COX7C; Abcam), S100 Calcium Binding Protein A11 (S100A11; Sigma-Aldrich) and glyceraldehyde 3-phosphate dehydrogenase (GAPDH) (Thermo Fisher Scientific; Abcam). After the incubation of the membranes with peroxidase-conjugated secondary antibodies (Life Technologies; Invitrogen, Waltham, MA, USA) (Table S5), signals were visualized through the LiteUP Western Blot Chemiluminescent Substrate (EuroClone). Images were acquired using the ChemiDocTM MP Imaging System (Bio-Rad), and densitometric analysis was performed with ImageLab software 6.0.1 (Bio-Rad). Proteins’ levels were normalized according to GAPDH signal.

### 4.7. Quantification of Active Mitochondrial Number and ROS Production

CMSCs were harvested and treated with 200 nM MitoTracker Green and with 5 μM MitoSOX Red (ThermoFisher Scientific). MitoTracker Green has been used for the quantification of cells with active mitochondria, MitoSOX Red for the quantification of mitochondrial superoxide production. Fluorescence of the samples was measured with the Amnis ImageStream^®^X Mk II device (Amnis Corporation, Austin, TX, USA) with 40× magnification and low flow rate/high sensitivity using the INSPIRE ImageStreamX MkII software. Data from 10,000 events per sample were collected, and the median of fluorescence intensity was calculated using the IDEA 6.2 software (Amnis Corporation).

### 4.8. Proliferation Analysis

The proliferation rate of CMSCs was tested using an EdU assay kit (Abcam), an index of DNA replication. CMSCs were seeded with a density of 2.5 × 10^4^/cm^2^ cells, and after 24 h, they were incubated with medium, supplemented with 40 μM EdU, at 37 °C for 4 h. Subsequently, CMSCs were detached, fixed with the fixative solution (4% formaldehyde-based) and permeabilized with the permeabilization buffer (Triton X-100-based). For EdU detection, cells were incubated with iFluor 488 azide and measured with the Gallios flow cytometer platform by using Kaluza 1.1 acquisition software (Beckman Coulter, Brea, CA, USA). Data from 5000 events per sample were collected and examined with Kaluza 1.3 analysis software.

### 4.9. Epicardial-to-Mesenchymal Transition Evaluation

EMT stage was evaluated by analyzing the protein expression of the selected epicardial markers ALDH1A1, TCF21 and WT1. ACM and HC-CMSC were harvested, fixed and permeabilized using the Fixation/Permeabilization kit (BD Biosciences, Franklin Lakes, NJ, USA). CMSCs were incubated with the proper primary and secondary antibodies (Table S5): ALDH1A1 (Abcam), TCF21 (Abcam), WT1 (Abcam); Goat anti-Rabbit 488 (Invitrogen). Fluorescence detection for 5000 events per sample occurred with the Gallios flow cytometer platform by using Kaluza 1.1 acquisition software, and the analysis was conducted using Kaluza 1.3 analysis software (Beckman Coulter).

### 4.10. Statistical Analysis

Quantitative results are expressed as mean ± standard deviation. Comparisons between groups were performed by Mann–Whitney test or Wilcoxon test on GraphPad Prism 9. Findings with *p* value ≤ 0.05 were considered statistically significant.

## Data Availability

The data supporting the findings of this study are available within the article and its Supplementary Materials. All other supporting data are available from the corresponding author on reasonable request. The transcriptome (ID: GSE233780) and methylome (ID: GSE234183) data are available under SuperSeries GSE234184 on the GEO repository.

## Supplementary Materials

The following supporting information can be downloaded at: https://www.mdpi.com/article/10.3390/ijms241210017/s1.
