# Supplementary material for: Omics Analyses of Stromal Cells from ACM Patients Reveal Alterations in Chromatin Organization and Mitochondrial Homeostasis
Source: Int J Mol Sci. 2023 Jun 12;24(12):10017. doi: 10.3390/ijms241210017 (PMC10298442; doi:10.3390/ijms241210017)
Supplement: Supplementary file 1 [file ijms-24-10017-s001.zip › Supplementary Table S4 and Table S5.pdf]

Supplementary Material

Sample ID	Sex	Age	Global or regional dysfunction and structural alterations	Tissue characterization of wall	Repolarization abnormalities	Depolarization abnormalities	Arrhythmic events	Family history	CMSC use
ACM1	M	57	dyskinesia, fibro-fatty substitution	fibro-fatty substitution	iTW in V1-V2	no	PVC	*PKP2*:c.544G>A (VUS)	methylome, transcriptome, WB, epicardial markers, proliferation
ACM2	M	42	dyskinesia, RV dilation, RV EF depression	n.c.	iTW in V1-V6	no	SVT	*PKP2*:c.2013delC (pathogenic)	methylome, transcriptome, epicardial markers, proliferation
ACM3	F	39	no	fibro-fatty substitution	iTW in V1-V2	epsilon waves in V1-V3	no	*PKP2*:c.2013delC (pathogenic)	methylome, transcriptome, WB, epicardial markers, proliferation
ACM4	M	41	bulging, RV dilation, RV FAC depression	n.a.	iTW in V1-V6	no	PVC	*PKP2*:c.2013delC (pathogenic)	methylome, transcriptome, WB
ACM5	M	52	dyskinesia	fibro-fatty substitution	no	no	SVT	*PKP2*:c.2013delC (pathogenic)	methylome, transcriptome, epicardial markers
ACM6	M	41	dyskinesia, RV dilation, RV EF depression, fibro-fatty substitution	fibro-fatty substitution	no	no	PVC	*PKP2*:c.548G>A (VUS)	methylome, transcriptome, WB, epicardial markers, proliferation
ACM7	F	41	no	fibro-fatty substitution	iTW in V1-V2	no	SVT, PVC	n.a.	WB, epicardial markers
ACM8	F	24	dyskinesia, BiV dilation, fibro-fatty substitution	n.c.	iTW in V1-V6	no	SVT	*DSG2*:c.1003A>G (VUS)	epicardial markers
ACM9	M	20	BiV dilation	myocyte loss, fibro-fatty substitution	iTW in V4-V6	no	NSVT, PVC	negative	epicardial markers
ACM10	F	52	BiV dilation	fibro-fatty substitution	no	no	PVC	*JUP*:c.1359G>T (VUS)	epicardial markers, proliferation
ACM11	M	52	dyskinesia, RV FAC depression	n.c.	iTW in V1-V2	no	syncope	negative	WB, epicardial markers, proliferation
ACM12	M	51	akinesia fibro-fatty substitution	fibro-fatty substitution	iTW in V4-V6	no	NSVT	negative	WB
ACM13	F	48	dyskinesia, RV FAC depression	fibro-fatty substitution	iTW in V4-V6	no	SVT	negative	WB
ACM14	M	69	dyskinesia, RV EF depression, fibro-fatty substitution	n.c.	no	no	PVC	negative	WB
ACM15	M	43	dyskinesia, RV FAC depression	myocyte loss, fibro-fatty substitution	iTW in V1-V3	no	SVT	negative	epicardial markers, proliferation

Table S4. Clinical table. Summary of the main clinical characteristics of the ACM patients. Abbreviations: iTW: inverted T waves; PVC: premature ventricular contractions; VUS: variant of uncertain significance; RV: right ventricle; EF: ejection fraction; n.c.: not conclusive; n.a.: not available; SVT: sustained ventricular tachycardia; BiV: biventricular; FAC: fractional area change.

	Antibody	Host specie	Type	Company
WB	Cyclin D2	Rabbit	Primary	Abcam
	Cytochrome c Oxidase 7C	Rabbit	Primary	Abcam
	S100 Calcium Binding Protein A11	Mouse	Primary	Sigma-Aldrich
	Glyceraldehyde 3-phosphate dehydrogenase	Mouse	Primary	Abcam
	Glyceraldehyde 3-phosphate dehydrogenase	Rabbit	Primary	Thermo Fisher Scientific
	Anti-Mouse peroxidase-conjugated	Goat	Secondary	Life Technologies
	Anti-Rabbit peroxidase-conjugated	Goat	Secondary	Invitrogen
FACS	Aldehyde dehydrogenase 1A1 PE-conjugated	Mouse	Primary	Abcam
	Transcription factor 21	Rabbit	Primary	Abcam
	Wilms Tumor Protein 1	Rabbit	Primary	Abcam
	Alexa Fluor anti-Rabbit 488	Goat	Secondary	Invitrogen

Table S5. Summary of the antibodies used for Western Blot (WB) and FACS analyses. 5
